# Supplementary material for: Evaluation of Transplacental Antibody Transfer in SARS-CoV-2-Immunized Pregnant Women
Source: Vaccines (Basel). 2022 Jan 10;10(1):101. doi: 10.3390/vaccines10010101 (PMC8778956; doi:10.3390/vaccines10010101)
Supplement: Supplementary file 1 [file vaccines-10-00101-s001.zip › vaccines-1488602-supplementary.pdf]

Evaluation of transplacental antibody transfer in SARS-CoV-2-immunized pregnant women

SARS-CoV-2 Surrogate Virus Neutralization Test (sVNT)

The neutralizing antibody was determined using the sVNT (SARS-CoV-2 Surrogate Virus Neutralization Test Kit, GenScript, USA) and was performed according to the manufacturer's instructions. Positive controls, negative controls, and all samples were diluted with sample dilution buffer (1:9) and mixed with diluted HRP-conjugated RBD solution at a ratio of 1:1. After a 30-minute incubation at 37°C, the control mixture and sample mixture were added to a 96-well plate pre-coated with recombinant protein ACE2. After another 15-minutes incubation at 37°C, the supernatant of each well was removed, and the washing step was performed. Finally, tetramethyl benzidine (TMB) substrate solution was added to each well and the stop solution was subsequently added after a 15-minute incubation in the dark; this turned the color of the well from blue to yellow. Absorbance at 450 nm of each well was immediately read in a microtiter plate reader (Molecular Devices, USA). The inhibition percentage was obtained by the following calculation:

$$\text{Inhibition \%} = \left(1 - \frac{OD450\ \ value\ of\ sample}{average\ OD450\ value\ of\ negative\ control}\right) * 100\%$$

SARS-CoV-2 Neutralizing Antibody Detection (B.1.617.2 Variant)

A SARS-CoV-2 neutralizing antibodies detection kit (AdipoGen Life Sciences, UK; Cat.: AG-48B-0007-KI01) was applied to test the neutralizing antibodies against SARS-CoV-2 (specific to B.1.617.2 variant) present in either serum or plasma, and the protocol was presented following the manufacturer's instructions. All samples and controls were diluted in wash buffer 1X (1:9) and then added to a 96-well plate coated with spike proteins (receptor binding domain, specific to B.1.617.2 variant). After one hour incubation at 37°C, the coated wells were aspirated and washed five times using 1X wash buffer. Following this, the diluted ACE2-HRP solution was added to each well and incubated for one hour at 37°C before the washing step was repeated. TMB substrate solution was added to each well, and then the stop solution was added to end the assay procedure. The O.D. at 450 nm was measured in a microtiter plate reader (Molecular Devices, USA). The presence of neutralizing antibodies against B.1.617.2 variant in either serum or plasma was determined by calculating the percent inhibition using the formula:

$$\text{Inhibition \%} = \left(1 - \frac{OD450\ \ value\ of\ sample}{average\ OD450\ value\ of\ negative\ control}\right) * 100\%$$

S1 Receptor Binding Domain IgG Antibody Detection

The S1 receptor binding domain IgG antibody was tested by RayBio® COVID-19 Human IgG ELISA Kit (RayBiotech, USA; Cat.: IEQ-CoVS1RBD-IgG-1). The procedure was followed by the manufacturer's instructions. All reagents and positive control were prepared as required in advance. The samples were diluted by 1:1499. Prepared positive control and samples were added to a 96-well plate coated with the SARS-CoV-2 S1 receptor binding domain protein, which combined with corresponding antibody in samples and control. After incubation of one hour at room temperature with gentle shaking, the wells were washed, and biotinylated anti-human IgG antibody was added and incubated for 30 minutes at room temperature. After washing away unbound biotinylated antibody, HRP-conjugated streptavidin was added to the wells. After 30 minutes of incubation at room temperature, the washing step was repeated. TMB substrate solution was added to the wells and the stop solution was then added after 15 minutes in the dark, changing the color from blue to yellow. The intensity of the color was measured at 450 nm afterwards (Molecular Devices, USA).
